# Congenital Melanocytic Nevi

**Published:** 2016-01-28

**Authors:** Janki Shah, Adam M. Feintisch, Mark S. Granick

**Affiliations:** Department of Surgery, Division of Plastic Surgery, Rutgers–New Jersey Medical School, Newark

**Keywords:** congenital melanocytic nevi, classification, malignant potential, treatment, surgical management

## DESCRIPTION

A 4-year-old girl presented with congenital melanocytic nevus on the right forearm.

## QUESTIONS

1. **What are congenital melanocytic nevi?**
2. **How are congenital melanocytic nevi classified?**
3. **What is the malignant potential of congenital melanocytic nevi?**
4. **What are the treatment/management options available?**

## DISCUSSION

Congenital melanocytic nevi are benign proliferations of cutaneous melanocytes that arise as a result of abnormal growth, development, or migration of melanoblasts. Affecting approximately 1% of newborns, congenital melanocytic nevi form between 5 and 24 weeks of gestation and are present at birth or become apparent within the first year of life.^1^ The appearance of congenital melanocytic nevi varies considerably on the basis of morphology, texture, location, and size. The nevi can be round or oval with smooth, well-defined borders, and the surface texture can be papular, rugose, verrucous, or cerebriform. Congenital melanocytic nevi can be distinguished from acquired nevi through histological analysis, as nevomelanocytes of congenital melanocytic nevi are unique and extend below the surface of the skin, can spread to the deep dermis, and can also exist in the subcutaneous fat, fascia, or muscle.^2^ Although initially a nevus may be light in color, flat, or hairless, it can become more pigmented, raised, and acquire long, coarse hairs. While they can appear anywhere on the body, the most common anatomic location for a giant congenital melanocytic nevus is the posterior trunk, followed by the legs, arms, head, and neck.^3^

Clinically, congenital melanocytic nevi are classified on the basis of their size. Small nevi are generally considered to be less than 1.5 cm in greatest diameter, medium nevi between 1.5 and 19.9 cm in greatest diameter, and large or giant congenital melanocytic nevi 20 cm or more in greatest diameter.^2^ In addition, because the growth of these lesions is proportional to overall growth, a more precise definition of giant congenital melanocytic nevi compares the size of the lesion with the total body surface area; lesions that occupy 2% or more of body surface area are classified as giant nevi. Giant nevi often have “bathing trunks” and “glove stocking” distributions and can appear with several smaller satellite lesions.^4^

The lifetime risk for malignant transformation of congenital melanocytic nevi depends largely on size. With high variability throughout the literature, generally accepted percentages toward development of melanoma range from 0% to 5% for small congenital melanocytic nevi, with one's risk increasing to approximately 5% to 10% for large congenital melanocytic nevi.^5^ When arising from a larger lesion, the melanoma is more likely to develop deep into the dermal-epidermal junction and may be more difficult to detect. Because 70% of melanomas developing from large congenital melanocytic nevi occur within the first decade of life, early excision of giant congenital melanocytic nevi compared with smaller lesions is essential. Features suggestive of dysplasia or malignant transformation to melanoma, including accelerated growth, ulceration, and changes in color, shape, or nodularity, should prompt a biopsy of the congenital melanocytic nevus, regardless of the initial size of the lesion.^4^^,^^6^

Treatment options currently available for congenital melanocytic nevi include observation, dermabrasion, chemical peel, laser ablation, and complete excision. The primary goal of management is to remove the risk of malignant transformation while minimizing functional deformity and improving cosmesis. Because of the low risk of malignant transformation, observation is often recommended for lesions not amenable to ablative or surgical therapy due to significant disruption of function with excision. While dermabrasion, chemical peels, and lasers improve cosmetic appearance, they are not effective in completely removing deeper nevus cells since they are superficial treatments. When treated superficially, these lesions require monitoring of recurrence and any residual nevus for signs of malignant transformation in the future.^7^ Only complete excision of the nevus with clear deep surgical margins can effectively reduce or eliminate the risk of malignant potential. Options for surgical management of congenital melanocytic nevi include 1-stage excision with primary closure, serial excision, tissue expansion, and excision with skin grafting. While serial excision is preferred when complete excision can be achieved in 2 to 3 stages, a combination of these techniques is often used on the basis of the anatomic location of the lesion. For example, in hair-bearing scalp, tissue expansion is the preferred method whereas full-thickness skin grafts are selected for lesions near the ear or eyelids, as serial excision here may result in deformity. In addition, because tissue expansion is linked to higher morbidity and failure rate in extremities, skin grafting may be useful for large lesions distal to the knee or elbow not amenable to serial excision.^8^

## Figures and Tables

**Figure 1:**
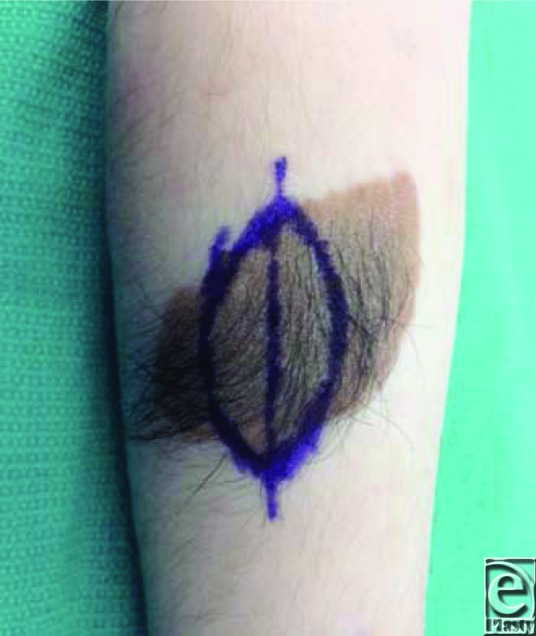
The surgical incision is marked for first-stage serial excision.
